# Spatial variations in the osteocyte lacuno-canalicular network density and analysis of the connectomic parameters

**DOI:** 10.1371/journal.pone.0303515

**Published:** 2024-05-14

**Authors:** Junning Chen, Marta Aido, Andreas Roschger, Alexander van Tol, Sara Checa, Bettina M. Willie, Richard Weinkamer

**Affiliations:** 1 Department of Biomaterials, Max Planck Institute of Colloids and Interfaces, Potsdam, Germany; 2 Department of Engineering, Faculty of Environment, Science and Economy, University of Exeter, Exeter, United Kingdom; 3 Julius Wolff Institute, Charité-Universitätsmedizin Berlin, Berlin, Germany; 4 Berlin-Brandenburg School for Regenerative Therapies (BSRT), Berlin, Germany; 5 Department of Chemistry and Physics of Materials, Paris-Lodron-University of Salzburg, Salzburg, Austria; 6 Department of Pediatric Surgery, Research Centre, Shriners Hospital for Children-Canada, McGill University, Montreal, Canada; The John Hopkins University School of Medicine, UNITED STATES

## Abstract

Osteocyte lacuno-canalicular network (LCN) is comprised of micrometre-sized pores and submicrometric wide channels in bone. Accumulating evidence suggests multiple functions of this network in material transportation, mechanobiological signalling, mineral homeostasis and bone remodelling. Combining rhodamine staining and confocal laser scanning microscopy, the longitudinal cross-sections of six mouse tibiae were imaged, and the connectome of the network was quantified with a focus on the spatial heterogeneities of network density, connectivity and length of canaliculi. *In-vivo* loading and double calcein labelling on these tibiae allowed differentiating the newly formed bone from the pre-existing regions. The canalicular density of the murine cortical bone varied between 0.174 and 0.243 μm/μm^3^, and therefore is three times larger than the corresponding value for human femoral midshaft osteons. The spatial heterogeneity of the network was found distinctly more pronounced across the cortex than along the cortex. We found that in regions with a dense network, the LCN conserves its largely tree-like character, but increases the density by including shorter canaliculi. The current study on healthy mice should serve as a motivating starting point to study the connectome of genetically modified mice, including models of bone diseases and of reduced mechanoresponse.

## 1. Introduction

As the descendants of osteoblasts, osteocytes function as the control centre modulating biochemical signals to orchestrate bone formation and resorption [1, 2]. Osteoblasts are trapped in osteoid and then differentiate into osteocytes when the matrix gets mineralized. In this process, osteocytes form a complex network with their numerous cytoplasmic processes which are believed to sense the mechanobiological stimulation [3, 4]. These processes, and the osteocyte cell bodies they belong to, are buried within the mineralized extracellular matrix (ECM) inside pores and channels known as the lacuno-canalicular network (LCN). The pervasiveness of the LCN in the bone allows it to deliver essential nutrients to osteocytes as well as remove waste products [2]. Recent evidence suggests that this intricate network also acts as communication channels with surface cells in a multicellular unit to regulate sophisticated cellular events [1, 2, 5].

Under dynamic loading, the deformation of mineralized ECM is believed to squeeze interstitial fluid to flow into and out of the LCN, passing through the pericellular space between the dendritic processes of osteocytes and the mineralized surfaces [6–8]. The surface traction stretches integrins that attach the osteocyte membrane to the extracellular matrix, therefore activating intracellular signalling pathways [6, 9]. Osteocyte lacunae are thought to serve as strain amplifiers [10], leading to nearly 10 times higher strains at the lacunar wall than on the tibial surface because of the stress-concentrating effects [11]. In addition, osteocytes are potentially involved in controlling the calcium and phosphate balance between ECM and interstitial fluid by resorbing their pericellular bone mineral [12–14]. Recent findings suggest that the LCN architecture is the result of an interplay between cells and surrounding ECM, closely associated with mechanical loading and indicative of mechanobiological activities and diseases [15–17]. The intimate link between the LCN architecture and functions could be interpreted as “connectome” [15], a term first introduced by neuroscientists to describe the pattern of connections between neurons [16]. This comprehensive approach to quantify the network architecture offers valuable insights in managing bone quality to provide the desired biomechanical properties and functions under various stimuli [17]. Moreover, it aids in understanding the mechanisms and alterations occurring in pathological conditions [18].

Mice have been used to study bone adaptation under mechanical loading on the tissue level, by combining *in-vivo* loading [17] with *in-vivo* microCT [19–21] and fluorescence labelling [18, 22]. Local bone formation and resorption were quantified to link with the local mechanical signals [23, 24]. It was found that mechanical loading has a stronger effect on enhancing bone formation than inhibiting resorption [20], and that the periosteal surface is less mechano-responsive than the endocortical surface of the tibia [25]. Since we and others [7, 26] have demonstrated that the LCN architecture is a determinant of the bone response to loading, asymmetries observed in (re)modelling activity [25] may in turn point to the architectural peculiarities of LCN. Despite the extensive number of studies examining individual osteocyte and its surrounding mineralized matrix [27–29], few studies have explored the LCN architectural characteristics across a larger scale to bridge a gap between the cellular and the tissue level [30, 31]. While the cortex of a mouse tibia primarily comprises lamellar bone, some regions of unordered woven-like bone remained from early bone development, contributing to its spatial heterogeneity [7, 32, 33]. In these regions, osteocyte lacunae appear without preferred orientation in the cross-sectional plane, but highly aligned along the long axis of the bone [34]. Considering heterogeneous biomechanical stimulation induced by external loading in these regions, a large-scale mapping of LCN will allow interrogation on its link to the mechanics along the long axis of bone. In our recent studies [35–37], imaging the LCN with confocal laser scanning microscopy (CLSM) after rhodamine staining was only the first step in the evaluation procedure. Using image analysis, the images were then transformed into a mathematical network consisting of nodes (lacunae and intersection points of canaliculi) and edges (canaliculi). The data of the LCN connectome can then be structurally characterised by quantifying its density and topology [36, 37].

The main objectives of this study are twofold: firstly, to quantitatively characterize the lacuno-canalicular network architecture in mouse tibiae with a focus on network density and connectivity; secondly, to explore the spatial changes of network architecture along the long axis of the tibia. In addition, we test the hypothesis that controlled *in vivo* mechanical loading influences the network connectomics in both newly formed and pre-existing bone. The left tibiae of the mice were subjected to two weeks of controlled *in vivo* loading, while the right tibiae served as an internal control, and experiencing only physiological loading.

After euthanasia, the mouse tibiae were prepared along the sagittal plane to expose the network architecture within posterior and anterior cortices for comparison, which experience the maximal compressive and tensile loading, respectively [38]. Double labelling with calcein at Day 3 and 10 allowed differentiating the LCN architecture between the mature bone and the bone newly formed in response to *in vivo* loading. This study, focusing on healthy mice, establishes an encouraging foundation for exploring the connectome affected by other physiological factors, including sex and age, as well as altered mechanical loading conditions. In addition, the osteocyte LCN of genetically modified mouse models representing various bone diseases can be investigated and compared to our current findings in healthy mice. This direction of research promises to deepen our understanding of mechanoresponse mechanisms and their relation to bone pathophysiology.

## 2. Materials and methods

### 2.1. Animal handling, loading and labelling

**Fig 1:**
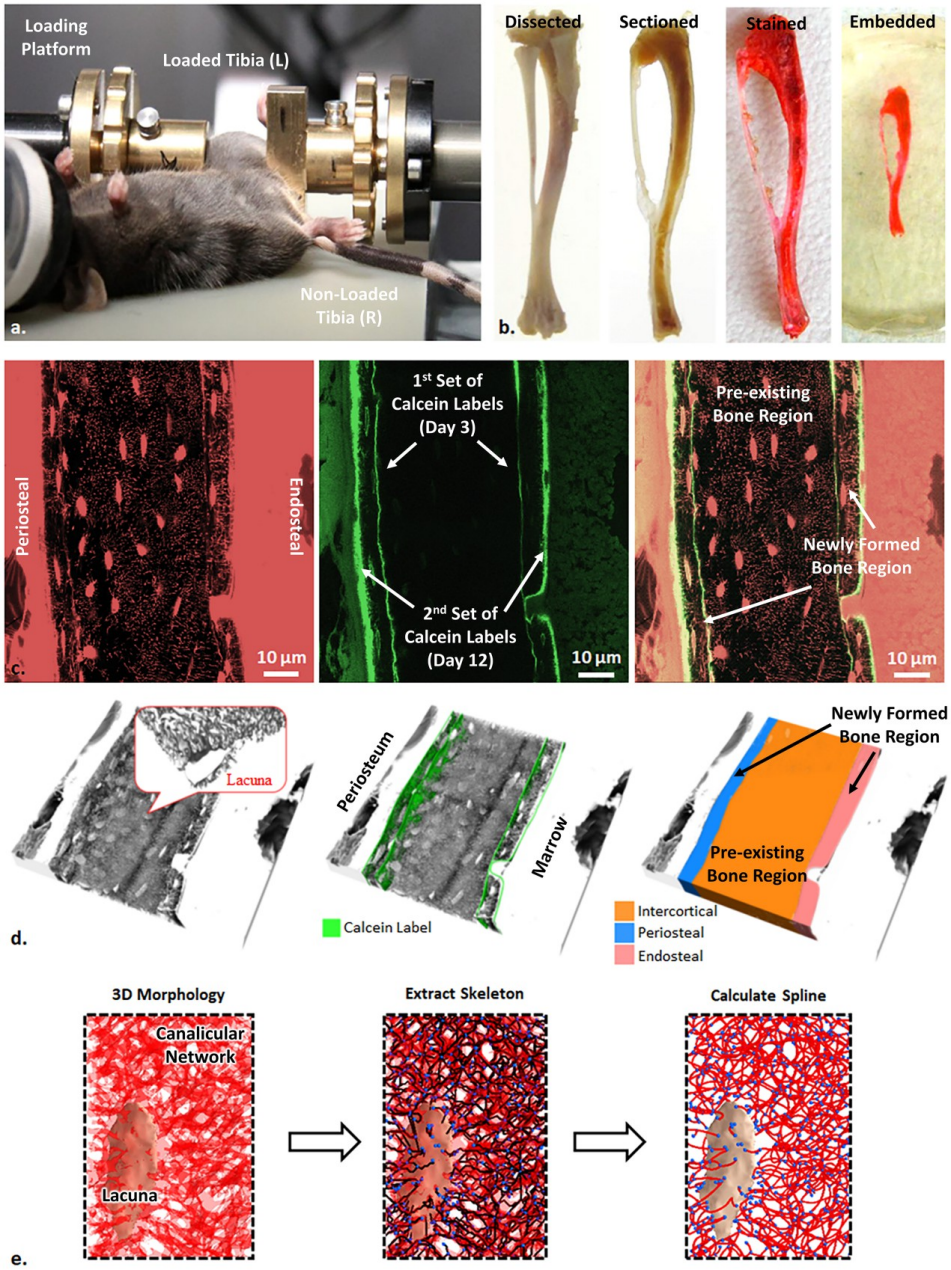
a) Experimental setup for non-invasive loading of left C57BL/6J mouse tibia and the right nonloaded control tibia; b) sample preparation for imaging of the osteocyte lacuno-canalicular network (LCN) with confocal laser scanning microscopy (CLSM) (left to right: tibia extracted and dissected from the subject, sectioned along the sagittal plane, stained with rhodamine, and embedded in PMMA); c) single-slice CLSM image using two isolated signal channels (red: rhodamine; green: calcein) and their fused image (right) of a loaded posterior cortex at the mid-shaft region, showing the osteocyte LCN in the region of more mature bone (between the 1^st^ set of calcein labels) and the osteocyte LCN in the regions of newly formed bone (between the 1^st^ and 2^nd^ set of calcein labels); d) 3D rendering of scanned osteocyte LCN in the volume and masking for different regions by using calcein double labels in 3D (orange: intercortical; blue: newly formed bone at the periosteal surface; pink: newly formed bone at the endosteal surface); e) an example of obtaining mathematical network description in a sub-volume with an osteocyte lacuna (beige almond-shaped object at the bottom left), by segmentation using adaptive thresholding to separate lacune and canaliculi, skeletonization and polynomial spline fitting to convert pixel-based data into a connectome network.

In our study, we chose to examine skeletally mature, 26-week-old female C57BL/6J mice, since previous research has shown a more pronounced mechanoresponse in female C57BL/6 mice compared to males [39, 40]. In addition, the reduced aggressive behaviour of female mice results in a lower background of physical activity, which makes detection of the bone’s mechanoresponse to in vivo loading much easier [41]. The animals were received (Jackson Laboratories, Sulzfeld, Germany) and acclimatized in our animal facility for two weeks prior to the onset of the experiment. Mice were group-housed in a cage with ad libitum access to water and a maintenance diet through a 12:12 hour light/dark cycle. Daily cyclic compressive loading (216 cycles/day at 4 Hz) was applied to the left tibia for 2 weeks (5 days/week, Monday-Friday) using an *in vivo* loading device (Testbench ElectroForce LM1, TA Instruments) (Fig 1a). Before and during loading, mice were anesthetized using isoflurane (2% in 2.0 L/min O_2_). The peak load in each cycle was -11 N, which is known to engender 1200 με at the medial surface of the midshaft in this mouse age/gender/genotype [42]. This strain level has been shown to elicit bone structural adaptation [20, 21, 43]. The loading cycle went through a triangle waveform, consisting of 0.15 seconds symmetric active loading/unloading and 0.1 seconds rest insertion (at 1 N). Between every four cycles, there was a 5 second pause. The right tibia was used as the control (non-loaded limb). On Day 3 and Day 12, three days prior to sacrifice, each mouse was administered an intraperitoneal injection of the fluorochrome label calcein (30 mg/kg). This was done to mark the active bone formation fronts between these specific time points. At day 15, the mice were euthanized through an overdose of potassium chloride while under anaesthesia (ketamine 60 mg/kg and medetomidine 0.3 mg/kg). All animal experimental procedures were approved by the local animal welfare representative (LAGeSo—Landesamt für Gesundheit und Soziales Berlin, G0333/09), and all experiments were performed in accordance with relevant guidelines and regulations. The work followed the ARRIVE Essential 10 guidelines in study design and reporting.

### 2.2. Sample preparation and imaging

A sample preparation protocol [36, 44] established through previous studies was adopted here. Both left (loaded) and right (control) tibiae (n = 6 tibiae) were harvested on Day 15 (Fig 1b). After clearing with xylene, samples were fixed in 100% ethanol. To allow thorough diffusion of rhodamine, the samples were cut open along a lateral plane, parallel and close to the sagittal plane, by using a diamond saw (IsoMet; Buehler GmbH, Düsseldorf, Germany) with a customized sample holder. Commercial PMMA (Technovit 9100, Heraues Kulzer, Wehrheim, Germany) was used for embedding. Rhodamine 6G (Sigma-Aldrich, Missouri, USA) was dissolved in both pre-infiltration and infiltration solutions (0.002% wt.), and the samples were soaked in the pre-infiltration solution for one day and the infiltration solution for 7 days. The stained samples were embedded in PMMA at 4°C for 48 hours and were ground and polished (Logitech PM5; Logitech Ltd., Glasgow, UK) until the tibia sagittal plane was exposed to the surface.

The open surface of each individual finished sample was first examined and mapped with a digital optical microscope (VHZ-100UT, Keyence, Itasca, U.S.A.) to identify the anatomical landmarks and orientation of the specimen. The midshaft region (40–55% of the overall tibial length—a region documented to have a robust bone formation response to *in-vivo* mechanical loading [17, 42]) was then identified and mapped under a confocal laser scanning microscope (Leica TCS SP5, Leica Microsystems GmbH, Wetzlar, Germany), with a 40X dry objective. The laser wavelength was set to 543 nm for rhodamine excitation and 488 nm for calcein, with each being activated sequentially. The corresponding photomultiplier tube (PMT) detector ranges were set from 550 to 670 nm and from 500 to 530 nm, respectively. The anterior and posterior cortices at mid-shaft regions were further scanned under a 100X oil immersion objective along the z-direction. The 100X lens provided a pixel size of 303 nm, but the high magnification limits the field of view. To extend the field of view each cortex image set was stitched from multiple z-stacks with 20% overlapping in both x- and y-directions, by using 3D-stitching plugins in Fiji ImageJ. Fig 1c shows the slices of stitched images acquired from the two channels and their superimposition. The red channel (left) indicates the osteocyte LCN captured, along with the soft tissues stained by rhodamine, such as bone marrow and periosteal membranes. The green channel (middle) presents the calcein labels produced at the active bone front after two injections. The superimposition of the images allow differentiating the osteocyte LCN in the more mature bone tissue (between the 1st set of calcein labels) and the osteocyte LCN in the newly formed bone tissue (between the 1st and 2nd set of calcein labels). The attenuation of fluorescent radiation was quite substantial. To ensure a robust signal from calcein, the scanning depth was limited to 30 μm and the fluorescence signal was compensated by increasing laser intensity and photomultiplier gain along the depth.

### 2.3. Image processing and segmentation

The raw image data was firstly masked to isolate the volume of interest for further analysis from surrounding soft tissues and embedding material. The volume was divided into three distinct regions based on the calcein labels, 1) the newly formed bone at the periosteal surface, 2) the intercortex comprising all the “old” pre-existing bone and 3) the newly formed bone at the endosteal surface (Fig 1d). The masked image stack was then evaluated using our in-house built software package, Tool for Image and Network Analysis (TINA). A detailed description of the algorithm and procedures has been included in our previous publications [35–37] and can be viewed on its main page (https://bitbucket.org/refelix/tina/).

In a nutshell, the image stack was first binarized employing a customized adaptive threshold algorithm based on the Difference of Gaussians (DoG) with two different sets of parameters to segment the thin canalicular structures and the bulkier lacunae. The binarized image was then skeletonized by removing the surface voxels, to form a topological network consisting of only the voxels along the centre lines of the morphology, as a thinned version of the original image (Fig 1e). As the final step, third order polynomial splines were fitted to this network as chains of voxels, so its discrete nature was mitigated with continuous edges connected through nodes in this network. The outcome of this procedure is a mathematical network defined by its nodes (i.e., lacunae or intersection points between canaliculi) which are connected via edges (i.e., canaliculi).

### 2.4 Canalicular network quantification

The network, comprising nodes and edges, was quantified using various topological parameters. Among all, three key parameters of the most interest to us, including the canalicular network density (Can.Dn), canalicular lengths (Can.Ln), and the degree of connectivity (DoC) of each node. The canalicular network density (Can.Dn) was calculated as the total length of the canaliculi (in terms of all canalicular segments) within the volume of interest, $\text{Can}.\text{Dn}=\frac{\sum Can.Seg_{i}}{V_{ROI}}$; and is, therefore, reported in μm/μm^3^. In the evaluation of Can.Dn, the volumes of lacunae and vascular canals were excluded. To examine the spatial variation of the network properties, the volume of interest (i.e. the full cortex) is further partitioned into sub-volumes with an edge length of 5 μm, resulting in a volume of 125 μm^3^. The choice of the size of the sub-volume has to be a compromise to be as small as possible to provide a balanced spatial resolution, but to be sufficiently large to provide robust values for the connectomic parameters. A convergence test on the sub-volume size was conducted to ensure the choice was not too big to average the local heterogeneity and gradient, or too small to represent the local volume. A range of edge lengths were tested from 1 to 20 μm for the sub-volumes. The distributions of the canalicular network density were consistent between 4 and 6 μm. In this study, 5 μm was adopted for the analysis, to provide clear variations in the newly formed regions and along the imaging depth (z-direction, the shortest dimension of the imaged volume).

To interpret spatial variations in the Can.Dn, we evaluate frequency distributions of the canalicular length (Can.Ln) and of the nodal degree of connectivity (DoC) in subvolumes, to assess the regional variations. Can.Ln was defined as the length of the canaliculus described by the 3D spline curve between two nodes in the network. A long and single canaliculus (i.e. network edge) can continue across multiple sub-volumes. When evaluating the length distribution in each of the sub-volumes, the sub-volume was considered for such a canaliculus, in which its centre of mass is located. The frequency distribution of Can.Ln was binned with a bin width of 1 μm to obtain a percentage distribution of the canalicular length. Canaliculi longer than 5 μm were considered as long canaliculi. Degree of connectivity (DoC) of a node in the network was defined by the number of edges connecting to this particular node. Per our definition, a node has to have at least three intersecting edges, i.e. the minimum degree is equal to three. Networks including only nodes of degree three are known as trees. Consequently, we refer to nodes of degree 3 as tree-like nodes. Similar to Can.Ln, the frequency distribution of DoC was normalized to obtain the percentage distribution of DoC. Spatial correlations were studied between the density of the network and the connectivity of the network characterized by the percentage of tree-like nodes (DoC = 3) and the percentage of long canaliculi (>5 μm), respectively.

This study was designed to examine the LCN connectome across extensive bone volumes, specifically whole cortex cross-sections imaged at high resolution, using a limited number of animals. As a result, we refrained from conducting statistical analyses between animals, such as assessing statistically significant differences by mean values. Instead, our focus was on assessing the variability of network parameters within the cortex, achieved by dividing it into thousands of sub-volumes. The data on the variability of the network density is shown in a box and whisker plot, indicating the median, 25% and 75% percentiles, and range of the variations in the sub-volumes. Reported values of the Can.Dn in the text correspond to the mean values across the specified regions of interest ± the standard deviation derived from three specimens included in this study. These values are calculated as the average and the standard deviation of all sub-volumes in a defined region of a specimen, for example, the anterior cortex of the loaded tibia of Subject 1 (M1).

## 3. Results

### 3.1 LCN density differences between anterior and posterior cortices

**Fig 2:**
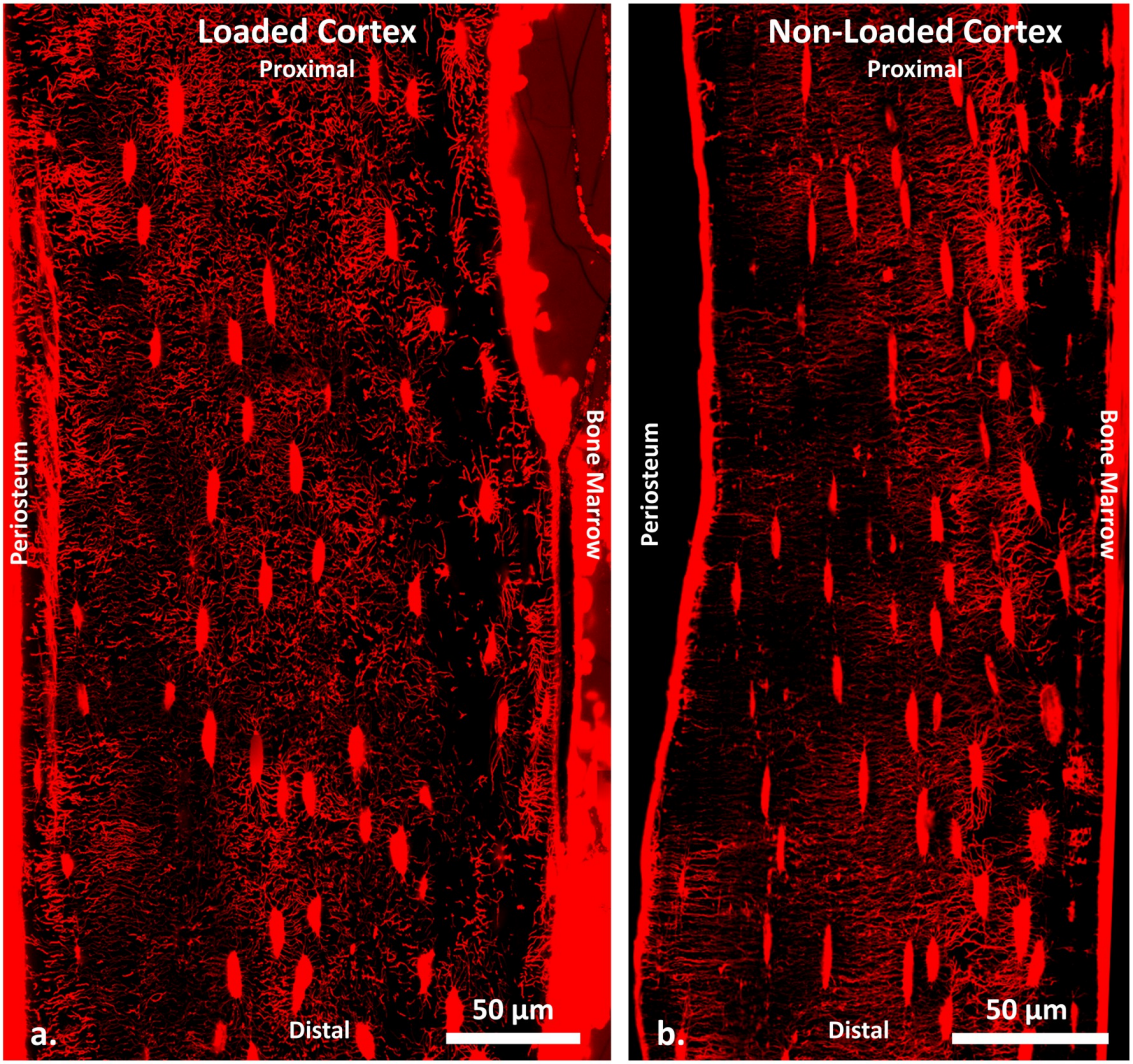
Projected confocal laser scanning microscopy (CLSM) stacks to show two representative data sets of the cortices under the loaded condition (a) and the non-loaded condition (b).

**Fig 3:**
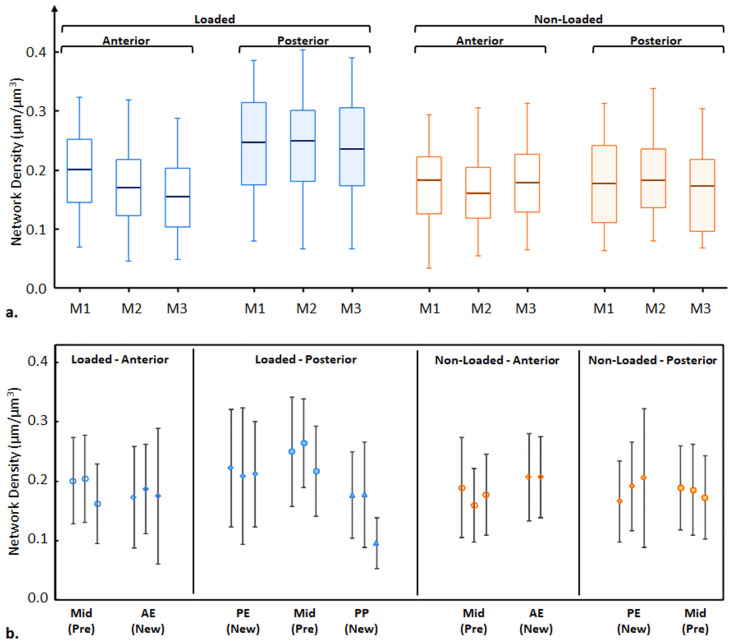
a) Box and whisker plot (indicating the median, 25% and 75% percentiles, and range of the variations in the sub-volumes) of canalicular densities (Can.Dn) from anterior and posterior cortices in the loaded (left) and non-loaded (right) tibiae from the three mice (M1-M3). b) Average canalicular densities (Can.Dn) evaluated in the newly formed regions (AE: Anterior Endosteal, PE: Posterior Endosteal; PP: Posterior Periosteal) and the pre-existing regions (Mid: Mid cortex). Note, we did not analyse the PP in the non-loaded tibia, nor the anterior posterior (AP) region in either the loaded or non-loaded tibia due to the limited amount of newly formed bone present.

Fig 2 presents two z-projected images showcasing the osteocyte LCN in representative loaded and non-loaded tibiae. These projections encompass only top sections (approximately 5 μm from a total depth of about 30 μm, similar to the sub-volume thickness adopted) to prevent overcrowding in the 3D to 2D representation. Within the limited depth of the tissue volume imaged (30 μm, about 110 slices), the acquired imaging data demonstrably reveals a marked heterogeneity in the distribution of network density across about 20 thousand of subvolumes within the cortices. This variability is particularly pronounced along the axis extending from the periosteal to the endosteal surfaces, indicating a gradient in structural organization and density that merits further analytical investigation. To quantitatively compare the global canalicular densities, Fig 3a compares the anterior cortex to the posterior cortex for both the loaded and non-loaded tibiae from all animals. To be noted, the mean and standard deviation of Ca.Dn in this work are derived from the reported regions of all three subjects in the specified regions of interest. All non-loaded controls show marginal differences between the anterior and posterior cortices on the canalicular network density (at -0.032, 0.013, and 0.014 μm/μm^3^), and the average density of all six regions of interest is 0.179 ± 0.013 μm/μm^3^. In the loaded tibiae, all three anterior cortices have similar LCN densities with an average of 0.176 ± 0.023 μm/μm^3^, also similar to the corresponding non-loaded controls (with differences at 0.011, 0.015, and -0.029 μm/μm^3^). The loaded posterior cortices exhibit higher canalicular density values than all other cortices in each subject (0.042, 0.072, and 0.066 μm/μm^3^ than loaded anterior, and 0.066, 0.054, and 0.051 μm/μm^3^ than non-loaded posterior/control), resulting in an average value of 0.236 ± 0.012 μm/μm^3^. However, based on the observed variations between each pair of samples, a substantially larger cohort is required to achieve robust statistical significance.

The fluorochrome labelling allowed us to differentiate the canalicular network in the regions of newly formed bone compared to the pre-existing bone region and to analyse their network densities separately Figs (1c & 3b). Our cyclic loading portfolio with a maximum load of -11 N is likely to result in lamellar bone formation, while other loading protocols, especially those engendering higher strain levels, can result in woven bone formation [17]. In the non-loaded (control) tibiae, there was only at the endosteal surfaces (AE—anterior endosteal, PE—posterior endosteal) sufficient new bone volume formed for analysis. C57Bl/6 mice achieve peak cortical bone density in the femur by 16 weeks [45] and whole bone strength in bending and torsion peaks by 20 weeks of age [46]. Thus at 26 weeks old, the mice have already reached peak bone mass and are already losing bone mass with increasing age [47]. In the loaded tibiae, all three mice have similar patterns of increased bone formation at the posterior cortex, while the anterior periosteal (AP) regions remained with very minimal growth. In the quantitative assessment of the LCN architecture, all three non-loaded controls showed little difference in the average LCN densities in the regions of the pre-existing (mature) bone (0.174 ± 0.020 μm/μm^3^) and the newly formed (endosteal-only) bone (0.184 ± 0.079 μm/μm^3^).

In the loaded posterior cortices, the newly formed bone in both periosteal and endosteal regions (PP and PE) have lower mean network densities (0.167 ± 0.056 and 0.214 ± 0.029 μm/μm^3^) than the pre-existing mid-cortex regions of the same three subjects (0.243 ± 0.053 μm/μm^3^). The establishment of the significance of this difference necessitates the inclusion of a larger sample size. In the controls, the difference between the newly formed and pre-existing bones was smaller with a trend of higher values of Can.Dn in the newly formed bone.

### 3.2 Spatial variation of the canalicular density across the cortex

**Fig 4:**
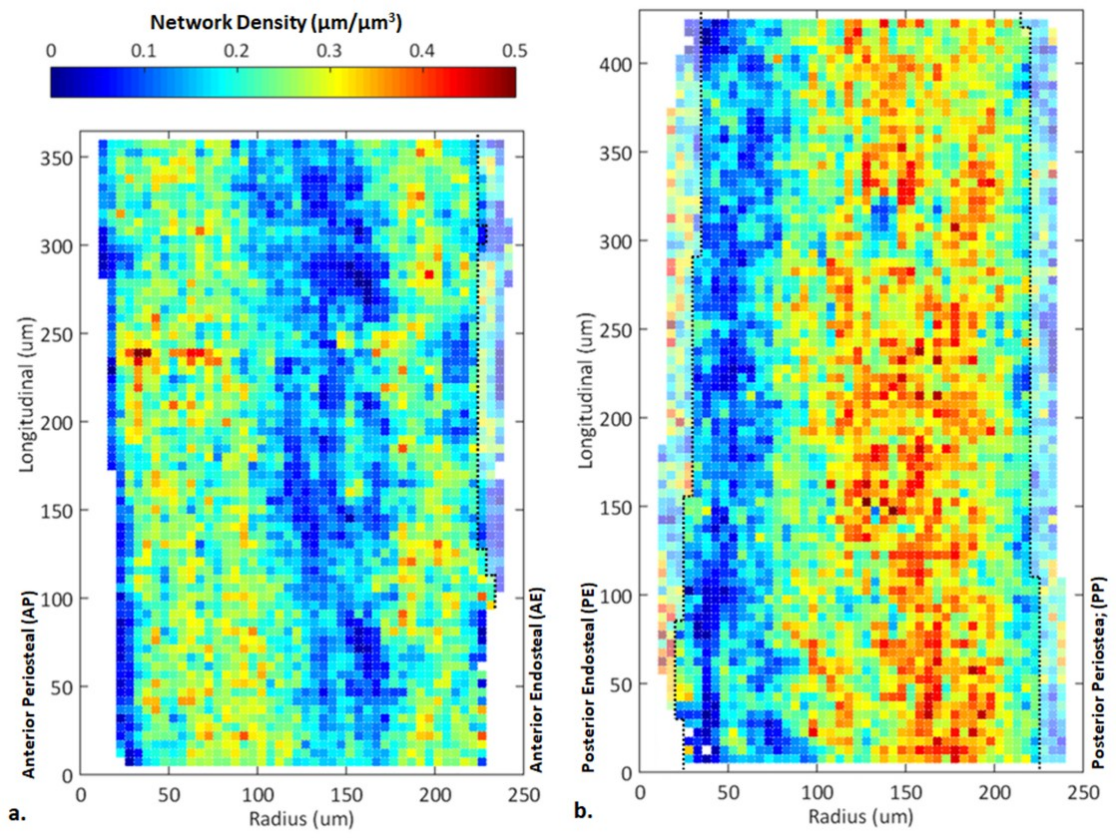
Colour maps of the canalicular density (Can.Dn) in the sagittal cross-sections of a) the anterior (partitioned into 3188 cubic sub-volumes in 3D with a side length of 5 μm) and b) the posterior (3714 cubic sub-volumes) cortices of the loaded tibia (representative example from mouse M1). Dark red corresponds to regions of highest canalicular density and dark blue indicates regions of the lowest density, according to the colour bar on top. Black dashed lines are defined by the first set of calcein labels (inner), to separate the newly formed bone (semi-transparent pixels) to the pre-existing region (solid pixels).

The substantial variability of the network density reflected by the large box sizes and whiskers of Fig 3 require an explanation in terms of a possible spatial pattern of this network parameter. To allow a characterization of the spatial heterogeneity of the LCN, the imaged volume was partitioned into smaller cubic sub-volumes with a side length of 5 μm. Fig 4 shows the network density maps in a representative pair of loaded anterior and posterior cortices. Dark blue (low network density) and red (high network density) regions in the plot differ by an order of magnitude in the canalicular density. In particular, the map highlights strong local gradients in the network density with neighbouring pixels having starkly different values of Can.Dn. The salient feature of these two maps is a coloured pattern of vertical stripes, which signifies that the variability in the canalicular density is rather low along the axis of the bone, but is large when moving across the cortex from the endosteal to the periosteal surface. This pattern is not symmetrical in the individual cortex or between the cortices. The blue stripes of lower network densities are closer to the endosteal surface.

**Fig 5:**
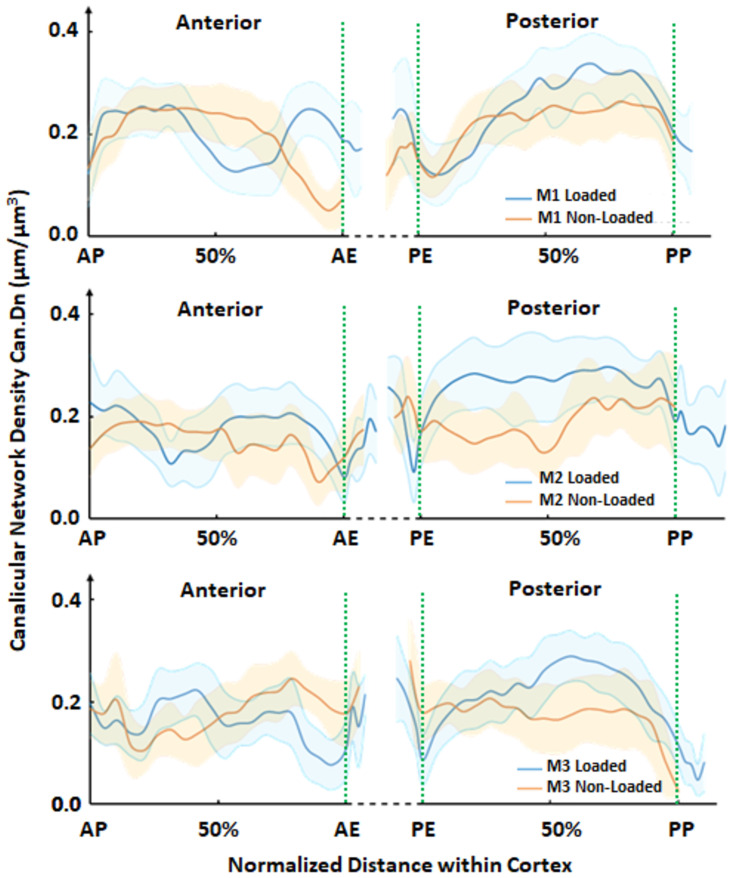
Loaded (blue) and non-loaded control (orange) spatial profiles of the canalicular density (Can.Dn) determined from thousands of sub-volumes, along the normalized length of the bone cortex from the endosteal to the periosteal region, with corresponding standard deviations in the shaded areas for all three mice (M1-M3). Anterior Periosteal (AP), Anterior Endosteal (AE), Posterior Endosteal (PE) and Posterior Periosteal (PP) specify the orientations. Green dashed lines indicate the locations of calcein labels and, therefore, indicate the boundary to newly formed bone.

Considering this stripe-like variability of the network density across the anterior and posterior cortices, the maps shown in Fig 4 were averaged along the longitudinal stripes to obtain spatial profiles of the canalicular density as a function of the position between periosteal and endosteal surfaces among all 3 mice in Fig 5. The vertical green dash lines in Fig 5 separate the pre-existing intercortical bone, from the newly formed bone (for AE, PE, and PP in the loaded, and AE and PE in the non-loaded cortices). The spatial profiles of the canalicular density show different locations of the maxima and the minima and are not symmetrical between anterior and posterior cortices.

In the posterior cortices of the loaded tibiae, the newly formed bone in the endosteal regions (blue lines in the PE regions separated by the green dash lines in Fig 5) tends to have increasing canalicular densities compared to the adjacent pre-existing intercortical bone. An opposite trend of decreasing values of Can.Dn of the new compared to the old bone were observed in the periosteal regions (blue lines in the PP regions separated by the green dash lines). There was no clear pattern in the canalicular densities when examining the profiles of non-loaded tibiae. Within the mature intercortical tissue, the posterior cortex of the loaded limb showed a trend of increasing canalicular density which decreased at the interface with newly formed tissue (blue lines at the green dash lines and nearby). The anterior cortices have a less homogeneous density distribution indicated by the wider shaded regions, and the patterns of the profile are more similar between the loaded (blue) and non-loaded (brown) specimens than at the posterior cortices. In all profiles, the network density tends to drop close to the boundary between “old” and newly formed bone (marked by the green dash lines), where the fluorochrome label is present (Fig 1c). Interestingly, we previously showed that regions in the bones of mice labelled with calcein fluorochrome have lower mean mineral thickness and degree of mineral alignment [48]. It should be noted that our study was not sufficiently powered to detect statistically significant differences between loaded and non-loaded limbs. Sample size analysis demonstrated that the strong heterogeneity found in the LCN density across the cortex, prevented us from drawing decisive conclusions based on our results between groups, due to the limited number of specimens.

### 3.3 Relationship between network density and network connectivity

**Fig 6:**
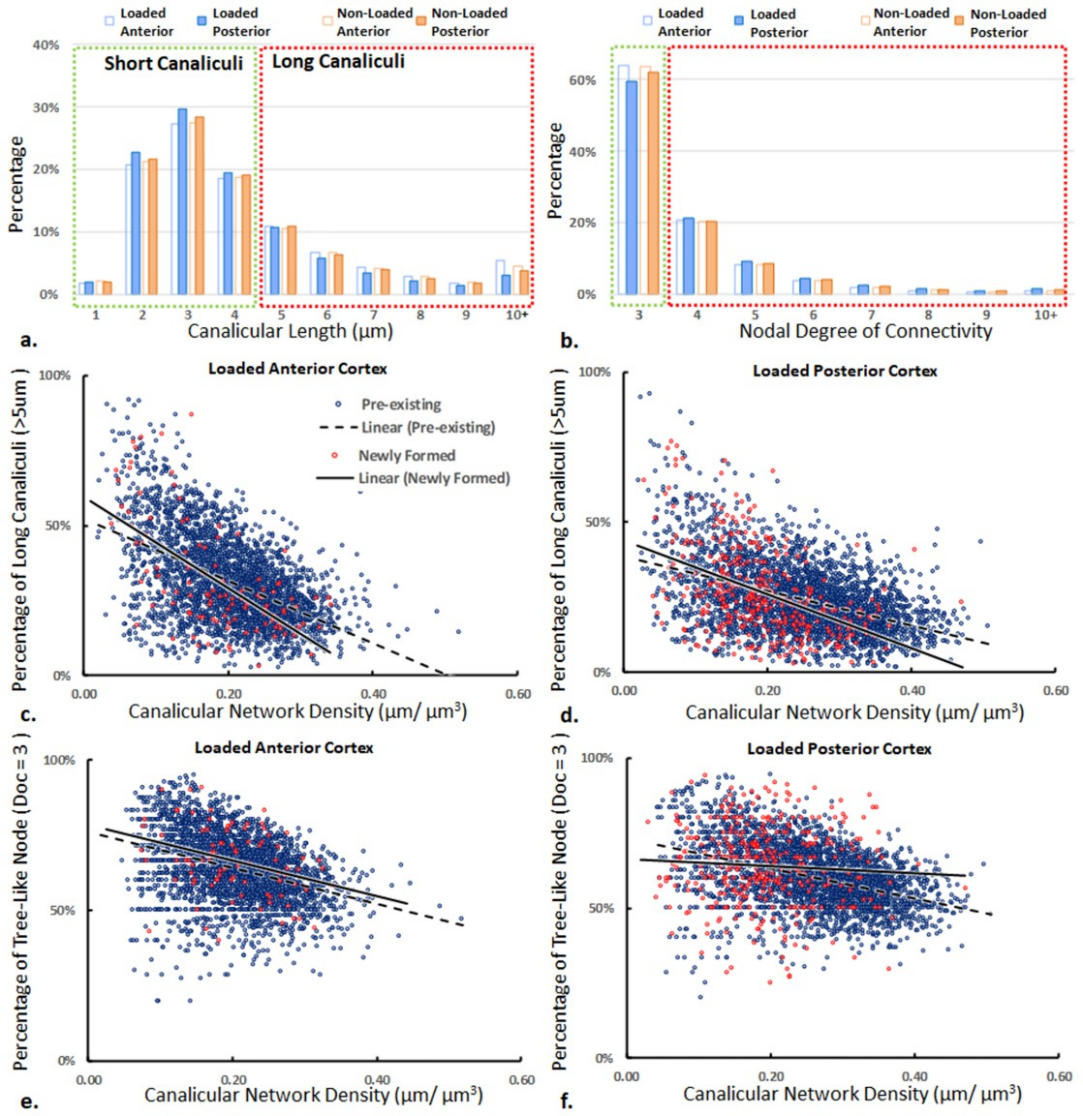
Normalized frequency distributions of a) canalicular length and b) nodal degree of connectivity in a representative loaded mouse tibia. Green boxes highlight more than 70% of the canalicular lengths and more than 50% of the nodal connectivity from the rest in their distributions. Scatter plot of the percentages of long canaliculi (c, d) and tree-like (DoC = 3) nodes (e, f) as a function of the network density in the corresponding anterior (left) and posterior cortex (right), respectively. Blue circles correspond to a sub-volume in the pre-existing intercortical bone, fitted with a dashed line using linear regression. Red circles correspond to sub-volumes in the newly formed tissue. Solid lines are the result of a linear regression and should only serve as a guide to the eye.

Beside the density of the network, we analysed the length of canaliculi connecting nodes in the network and the connectivity of these nodes. Both analysis were conducted on all specimens included in this study, and Fig 6a and 6b show the representative percentage distributions of canalicular length and nodal degree of connectivity (DoC) in the loaded anterior and posterior cortices of M1 as the examples. Overall, more than 70% of the canaliculi (highlighted in the green dash box) are less than 5 μm in length, and tree-like nodes (DoC = 3, the minimum degree) are dominant in all cortices (more than 50%, as highlighted in red boxes). The posterior cortex tends to have more short canaliculi and fewer tree-like nodes than the anterior cortex both in loaded and non-loaded tibiae.

To correctly interpret variations in the canalicular density, we asked the question if the large values of the canalicular density found in certain regions are (i) due to a greater branching of the network; or (ii) are related to the length of the canaliculi. Fig 6c and 6d show the (length) percentages of long canaliculi as a function of the network density in the anterior and posterior cortices for a representative loaded tibia, and Fig 6e and 6f shows the percentages of the tree-like nodes in the corresponding cortices. Although the data scatters substantially (R^2^ < 0.4), there is a clear trend that the prevalence of both long canaliculi and tree-like nodes decreases with increasing canalicular densities. Such negative correlations demonstrate that the regions with a dense network are characterised by a more branched network with shorter canaliculi. However, the slopes of the linear regression show more negative values for the percentage of long canaliculi (ranging from -1.41 to -0.29) compared to tree-like nodes (ranging from -0.58 to -0.12). Even in the densest parts of the network, most of the nodes still has the tree-like connectivity (DoC = 3). Therefore, while denser parts of the network have a trend towards higher connectivity, the dominant factor of network densification is to shorten the length of canaliculi while keeping the network architecture tree-like.

## 4. Discussion

Our study offers a quantitative assessment of the osteocyte lacuno-canalicular network architecture in mouse tibiae. On average, the canalicular network density in mouse tibiae is about 0.202 μm/μm^3^, which is equivalent to about 200 km of the network within a cubic centimetre of the tissue volume (i.e. 202 km/cm^3^). This value is nearly three times the reference value reported in human osteonal bone (0.074 ± 0.015 μm/μm^3^) [36] and is comparable to values found in the fibrolamellar bone of sheep [37]. One must put special emphasis on the average nature of this value since the network distribution varies substantially in the tissues. Low values of the network density are most likely related to locations of unordered matrix organization in the mouse cortex [49–51]. Meanwhile, each individual cortex exhibits a strong.

One key aim of this study is to advocate for a network-based perspective (connectomics) on bone microstructure, enabled by recent advances in imaging technology. While in the neurosciences connectomics was successfully adopted and described for a broader audience [52], it is still in its infancy for bone research [15]. There are two networks to be studied in bone, one inside the other: the cell network of osteocytes and the lacuno-canalicular network of pores and channels which can both be visualized simultaneously with staining [53]. In our approach, image stacks of the stained lacuno-canalicular network were transformed into network structures consisting of hundreds of thousands of nodes and edges [35, 36]. The analysis of the connectome potentially allows bridging the gap between the structure and the poorly understood multiple functions of the osteocyte network. In this study, we investigated the relationship between the network density, connectivity and canalicular length. In regions of a high canalicular density, the degree of the nodal connectivity is increased, but the majority of nodes remain with the minimal degree of three manifested as a tree-like branch structure. The more important structural change are the many short canaliculi that can be found in the dense network regions, as shown in Fig 6b at different anatomical locations of the cortex. Among the nodes, ones with a degree larger than six are probably identical with canalicular junctions, and in average 6.8 ± 2.6 canaliculi were reported meeting in a canalicular junction [54]. These junctions are distinctly smaller in volume (1–30 μm^3^) compared to lacunae (100–250 μm^3^).

In addition, this study on the LCN connectome was also designed to probe the influence of mechanical load on the network architecture. The mice underwent controlled *in-vivo* loading applied to the left tibia, while the right tibia served as a control with physiological loading. The cutting plane is along the gradient of the bending moment resulting in a maximal tensile stress in the anterior cortex and a compressive stress in the posterior cortex. The sagittal sections we adopted for this study maximizes the portion of newly formed bone in the images, which can be identified with calcein labelling. The maximum bone apposition is in the posterior region is similar to the findings in previous studies [20, 21]. The amount of newly formed bone in the adult mice after two weeks of *in vivo* loading is rather limited compared to the pre-existing intercortical bone. The analysis of the network architecture in the newly formed bone did not reveal clear effects from mechanical loading compared to mature tissue. We are cautious to state that mechanical loading has no influence on the LCN architecture. This caution is due to experimental limitations, which constitute challenges also for future studies.

The disadvantage of sagittal sections is that the cutting plane in long bones of mice is not perfectly reproducible and, therefore, varies between different animals. The variability of the profiles of the network density obtained from the individual mice (Fig 5) is likely attributable to small variations in the cutting plane. The rhodamine staining of the network has some limitations. Rhodamine enters in all the accessible canaliculi and attaches to the wall of mineralised extracellular matrix, to be captured with confocal laser scanning microscopy. Canaliculi are too narrow to be resolved by optical microscopy. The blurred rhodamine signal hinders determining the volume fraction of the LCN, and would result in a gross over-estimation than the values reported in the literature [28]. In fully mineralized bone, our algorithm was able to separate canaliculi in close vicinity and evaluate the density as the length/volume ratio through a reliable network analysis. In case of the newly formed bone with its lower mineral content, rhodamine enters deeper into the tissue, the signal become more blurred and, consequently, also the network analysis becomes more challenging.

The network density will likely strongly depend on the amount and type of newly formed bone resulting from the loading. Previous studies using *in-vivo* strain gauging and finite element analysis have shown that the loading protocol used in the current study engenders +1200μɛ on the medial mid-shaft (50%) of the tibia of adult female C57Bl/6 mice. This strain magnitude results in cancellous and cortical lamellar bone formation and does not elicit woven bone formation, which is usually observed when loading with higher strain magnitudes [17]. Woven bone in a fracture callus was reported to have 100% more osteocyte lacunae than cortical lamellar bone [55].

Our study also touches the question whether the architecture of the LCN is dynamic even in mature bone and still can adapt to mechanical loading. We found that when comparing the old bone (i.e., bone which was present before the *in-vivo* loading was applied), the loaded posterior cortices exhibited higher values than other regions in loaded and control tibias. Although this was the case for all three mice, due to the low sample number we cannot claim a statistical difference between the density of the LCN in loaded and unloaded bone. It has been reported that osteocytes occupy dynamically the LCN by moving their cell processes into and out of the canaliculi as well as movement of their cell body [56–58]. What is unclear is whether also the architecture of the LCN changes with time by removal and creation of canaliculi. Observations of network-free regions in human osteons [36] suggest that the network structure may deteriorate, most likely by clogging of some of the canaliculi by micropetrosis [36, 59]. The creation of new canaliculi seems harder to imagine, however, the evidence of Tartrate-resistant acid phosphatase (TRAP) activity—a biochemical marker of bone resorption—in osteocytes could open a possibility in this direction [60].

The profound heterogeneity found in the LCN density across the cortex limits our ability to conduct hypothesis testing, due to the large sample size required. Transversal sections of mice tibiae and femora from recent studies show that the strongest variations in heterogeneity are due to a ring of woven-like bone, which runs eccentrical within the cortex and is a remnant of early bone development^8,35^. Hence, we want to raise awareness on typical challenges and provide recommendations when it comes to the investigation of local properties with substantial heterogeneity such as the LCN density in bone: (i) In order to gain additional information to the mean values of connectomic parameters, frequency distributions are useful. (ii) A balance between sufficient sample number and in-depth quantitative analysis must be found when studying LCN connectomics. A sample size analysis using network density measured from matched limbs (left versus right) of mice from our study indicated 18 mice would be required to reject the null hypothesis. The large variability in network properties brings challenges to studying LCN in mouse models. Thus, in the present study we have performed an in-depth, comprehensive quantitative characterisation of the mouse tibia LCN connectome in a small number of mice. (iii) While longitudinal sections have the advantage of exposing a maximal area of newly formed regions at the periosteal and endosteal surface, they have the problem that the cutting plan is difficult to control. Individual differences in how the ring of woven-like bone runs through the cortex, does not provide robust results in terms of mean network properties, even with a perfect control of the cutting plane. This situation improves when looking at transversal sections at a well-defined location and if the measurement time is invested into mapping the whole cortex. (iv) The LCN density heterogeneity of the cortex outlines that there is no spatially limited anatomical location, which could be defined as “representative” for the whole bone. This underlines the importance of 3D methods with high spatial resolution. These would require additional investigation and serve as the aims for future studies. In addition, this connectomics approach could be applied to investigations on many other factors and scenarios. These include trabecular bone with higher topological complexity, different bone types with distinct biomechanics demands (e.g. flat bone), the effects of sex and ageing, and genetically modified mouse models reflecting pathological changes to deepen our understanding of bone pathophysiology and mechanoresponse mechanisms via quantitative characterization. Analogous to intensive efforts in brain research, the ultimate goal of a connectomics approach is to relate the LCN architecture with the multiple functions of the network. Parallel to this more basic science question, would be the development of a diagnostic tool, which allows an interpretation of network differences in terms of bone diseases.
